# Sanitation Versus Tuberculin in the Eradication of Tuberculosis

**Published:** 1898-06

**Authors:** G. N. Kinnell

**Affiliations:** Pittsfield, Mass.


### THE JOURNAL

OF

### COMPARATIVE MEDICINE AND VETERINARY ARCHIVES.

Vol. XIX. *JUNE, 1898.* No. 6.

### SANITATION VERSUS TUBERCULIN IN THE ERADICATION OF TUBERCULOSIS.

By G. N. Kinnell, M.R.C.V.S.,
PITTSFIELD, MASS.

As bearing on the question of eradicating tuberculosis from a diseased herd and of the competent disinfection of a tuberculosis-infected building, I submit reports of my experience in two cases, an experience which has stood the test of the past four years, and where the conditions which obtain to-day are, to all appearance and in every probability, entirely satisfactory.

First, we will take the herd of Mr. W. D. Sloane, of Lenox. In December, 1894, this herd comprised the following animals:

| | |
|---|---|
| Mature milch cows | 22 |
| Mature bull | 1 |
| Young bull | 1 |
| Young stock (heifers ranging from six months to two years old) | 11 |
| Total | 35 |

On Christmas day, December 25, 1894, the twenty-two milch-cows were submitted to the tuberculin-test, and the temperature-chart of the reactions obtained is herewith subjoined.

Chart 1.—Milch Cows.

| Herd number. | | | | | |
|---|---|---|---|---|---|
| 42 | 102° | 102.6° | 102° | 101.8° | |
| 30* | 102.6 | 106.6 | 107.4 | | |
| 34 | 102.12 | 102.4 | 102.4 | 102.4 | 102.2° |
| 45* | 101.4 | 105.8 | 104.2 | 105.4 | |
| 40* | 101.5 | 102.6 | 104.6 | 106 | |
| 28* | 100.8 | 103.2 | 103.2 | 104.8 | |
| 14* | 101 | 103 | 105 | | |
| 44 | 101 | 102.6 | 101.8 | 101 | 100.8 |
| 39 | 101.25 | 101.4 | 101.4 | 101.6 | 102 |

| Herd number. | | | | | |
|---|---|---|---|---|---|
| 4* . | . 100.8 | 102.4 | 102.6 | 104.6 | |
| 25* . | . 101.4 | 104.2 | 106.2 | | |
| 22* . | . 101.25 | 104.2 | 106 | 106.2 | |
| 7* . | . 100.6 | 102.4 | 103.6 | 105.4 | |
| 43* . | . 102.75 | 103.4 | 103.8 | 105.6 | |
| 19 . | . 100.6 | 102.4 | 102.6 | 102 8 | 102 |
| 21* . | . 101 | 106.2 | 106.6 | | |
| 38* . | . 101.4 | 104.4 | 106.4 | 107 | |
| 17 . | . 101.6 | 103 | 102.8 | 101 | 101.6 |
| 37 . | . 101.2 | 102 | 101.8 | 101 | |
| 48 . | . 101.5 | 103.4 | 101.8 | 101 | 101 |
| 49 . | . 102 | 103 | 102.6 | 101 | |
| 36* . | . 100.4 | 105 | 106.6 | 105.8 | |

The reagent used was the tuberculinum Kochii of Libbertz, the dose used being two minims diluted with 1 per cent. solution carbolic acid in distilled water. The column of figures to the left of the above chart are the herd numbers of the cows, and these numbers will again be referred to further on in the report.

It will be noticed that those animals marked with an asterisk (*), thirteen in all, gave decided reactions. Please note, also, that the diseased animals were in clumps or bunches. The worst-diseased individuals were found in the centres of these clumps. The animals were condemned by the State authorities and killed. In every instance well-marked tuberculosis was present, some of the cases being very bad indeed.

On January 20, 1895, all the young stock and both the bulls were tested; also five of the milch-cows were re-tested, and the following are the charts of temperatures obtained :

CHART 2.—TEMPERATURE OF YOUNG STOCK (HEIFERS).

No reactions.

| Herd number. | | | | | |
|---|---|---|---|---|---|
| 50 . | . 101.2° | 101.2° | 101° | 100 8° | 101.2° |
| 51 . | . 101 | 101.2 | 101 | 101 | 101 |
| 52 . | . 100.8 | 101.4 | 101.6 | 101.2 | 101.2 |
| 53 . | . 101 | 101.4 | 101 | 100.8 | 101 |
| 54 . | . 101 | 102 | 102.4 | 101.4 | 101.6 |
| 55 . | . 101.2 | 102 | 102 | 101.8 | 101.6 |
| 56 . | . 100.6 | 102 | 101.8 | 101 | 101.4 |
| 57 . | . 101.6 | 102.6 | 102.4 | 101.8 | 101.6 |
| 58 . | . 101.6 | 102 | 101.4 | 101.4 | 101.6 |
| 59 . | . 101.2 | 102 | 101.8 | 101.2 | 101.4 |
| 60 . | . 102.2 | 101.6 | 101.6 | 101.4 | 101.6 |

CHART 3.—TEMPERATURE OF BULLS.

| | | | | | |
|---|---|---|---|---|---|
| Young bull | 101.6° | 102° | 101.6° | 101.8° | 102° |
| Mature bull* | 101.2 | 103.4 | 106.2 | 106.8 | |

CHART 4.—TEMPERATURE OF RETESTED COWS.

Herd numbers the same as in Chart 1.

| Herd number. | | | | | |
|---|---|---|---|---|---|
| 44 . . | 100.8° | 101.4° | 101.6° | 101.2° | 100.8° |
| 39 . . | 101.4 | 101.2 | 101.8 | 101.6 | 101.2 |
| 19 . . | 102 | 102 | 101.8 | 101.4 | 101 |
| 17 . . | 101 | 101.2 | 101.2 | 101 | 101.4 |
| 48 . . | 101 | 101.6 | 101.6 | 101 | 101 |

It will be noticed that the only animal to react was the mature bull. He was condemned and killed. The lesions found were quite extensive, and, in my opinion, were of at least two-years' standing.

We had thus slaughtered thirteen out of twenty-two milch-cows and one bull, making fourteen out of a herd of thirty-five head.

All of the heifers were sired by the diseased bull, which must have been diseased at time of service. The dams of these heifers are shown by the following chart, and by reference to the first temperature-chart it will be seen that seven of these healthy heifers were born of diseased dams.

CHART SHOWING DAMS OF HEIFERS.

Dam of heifer No. 50 was cow No. 7* (tuberculous).
" " " " 51 " " " 21* "
" " " " 52 " " " 25* "
" " " " 53 " disposed of previous to test.
" " " " 54 " cow No. 28* (tuberculous).
" " " " 55 " " " 30* "
" " " " 56 " " " 21* "
" " " " 57 " " " 19 (healthy).
" " " " 58 " disposed of previous to test.
" " " " 59 " cow No. 37 (healthy).
" " " " 60 " " " 14* (tuberculous).

Mr. William Griffin (manager for Mr. Sloane), being anxious to make the work as thorough as possible, gave me *carte blanche* to kill any of the remaining animals which I might think looked in any way suspiciously of being diseased. Acting on this, we slaughtered the milch-cows Nos. 17–19 and 39. The two latter, Nos. 19 and 39, were found entirely clean and healthy in every way. In No. 17, on the other hand, we found a bronchial lymphatic gland enlarged to the size of a duck's egg, and containing glairy, liquid pus. There was an absence of the cheesy matter usually associated with tuberculosis, and the walls of the pus-cavity were thinner than we usually see in lymphatic glands undergoing tubercular suppuration.

It here devolves to say a few words on the arrangement of the stable where these animals were kept and on the steps taken to cleanse and disinfect it. While all the animals were kept under one roof, yet the building can best be described as consisting of two parts. That part in which the milch-cows were kept measures 30 x 60 feet, and the ceiling is $10\frac{1}{2}$ feet high. The floor is of brick, set on edge in cement. The windows, twelve in number, face east and west; they measure $3\frac{1}{2}$ x $2\frac{1}{2}$ feet, and are placed within two feet of the ceiling. There are two ventilators, each three feet square. The ceiling is of narrow pine boards, planed and varnished, and the lining of the walls is of the same material. The stable is arranged for twenty-six cows, the animals standing in two rows facing each other, with walks between and behind them about five feet wide.

The young stock were, for the most part, kept in box-stalls arranged in two rows and communicating with the cow-stable by a five-foot passageway. While under the same roof they were, with the exception of the passageway, isolated from the part in which the cows were kept, and, although reared on their milk, never came in direct contact with them.

*Cleansing and Disinfection.*

All the remaining stock having been removed, cleansing and disinfection was proceeded with as follows:

1. Dry brushing of the whole interior of the building.

2. Scrubbing the entire interior with soap and hot water *ad libitum.*

3. Saturation of the entire interior with an antiseptic wash made up in strength and of ingredients as follows: Bichloride of mercury, one ounce; glacial carbolic acid, twelve ounces; hot water (scalding), twelve gallons. This was applied copiously and forcibly with large garden syringes.

4. The doors and windows being shut tight, the atmosphere was saturated with the fumes of burning sulphur for a period of two nights and one day.

5. The doors and windows were then thrown open to the light and the air, and left so for a week.

6. The place was dried, the entire woodwork sandpapered and submitted to two coats of hard varnish.

7. And to all this I would say the work was done religiously and well.

In the bull-pen there was an extra lining of heavy, rough boards. These were removed and burned, but apart from this none of the woodwork was either removed or destroyed. In order to avoid danger of poisoning from the bichloride of mercury solution, the mangers were again washed out with simple warm water.

The animals were then taken back into the stable, and the herd replenished with tested cows from the farm of Mr. W. K. Vanderbilt on Long Island. Since then the herd has been self-sustaining. The original young stock as they developed have been introduced into the dairy, and other young stock have been born and grown up to take their proper place among the milch-cows, but during all these years no symptoms or evidences of tuberculosis have developed or been found either among the original or among the introduced stock. This negative evidence must be admitted as of some value, but apart from it we have, as the years went past, been able to accumulate a large amount of positive evidence which is of much greater value as a proof that the herd is free from disease. Thus during the last three years four[1] of the cows have been killed for beef, and inspection at time of slaughter failed to reveal any of the lesions of tuberculosis. In the month of September, 1897, seven of the milch-cows died from or were killed on account of poisoning with white lead. In all these cases a careful postmortem examination for the lesions of tuberculosis was conducted, but no lesions were discovered. The following table shows the animals that have been killed or have died.

TABLE SHOWING DISPOSAL OF THE BALANCE OF THE ORIGINAL HERD UP TO THE PRESENT TIME.

| | |
|---|---|
| Mature Cows. | |
| No. 42. | Still in herd. |
| " 34. | Killed for beef; healthy. |
| " 44. | Not in herd; no record of what became of her. |
| " 37. | Still in herd. |
| " 48. | Killed for beef; healthy. |
| " 49. | Sold to W. W. Law, of New York. |
| Heifers. | |
| No. 50. | Still in herd. |
| " 51. | Died of lead-poisoning September, 1897; not tuberculous. |
| " 52. | Killed for beef; healthy. |
| " 53. | Killed for beef; healthy. |
| " 54. | Died of lead-poisoning September, 1897; not tuberculous. |
| " 55. | Died of lead-poisoning September, 1897; not tuberculous. |
| " 56. | Still in herd. |
| " 57. | Still in herd. |
| " 58. | Still in herd. |
| " 59. | Still in herd. |

[1] Two of these were members of the original herd of mature cows.

Heifer.
No. 60. Still in herd.
" 61. Sold; no record of what became of her.
Nos. 51, 52, 54, and 55 were born of diseased mothers.

The second case I wish to point out was on a much smaller scale, and will not take so long to relate.

In June, 1894, Dr. Henry Colt, of Pittsfield, had six cows and a six-months'-old calf. The tuberculin-test was applied, and all of the cows reacted, the calf alone failing to do so. The cows were condemned and killed. Two of them proved to be unusually bad cases; one of these, a Jersey, was the mother of the six-months'-old calf, and was also pregnant at the time of slaughter.

The stanchions were removed and the floor, which was old, torn up and destroyed. The urine-soaked earth was dug up and removed. The interior was then brushed, washed and treated with the antiseptic wash previously mentioned. The ceiling and walls, being of rough, unplaned wood, were heavily washed with hot whitewash. The stable was left vacant and open to the sun and the air until the fall, when a new floor was put in, and the stanchions, which had been washed and left out of doors all summer, were put back in place. The calf was taken back into the stable, and has spent her winters there ever since. She is now a nice, plump four-year-old cow, and on January 12th of this year was tested with tuberculin without giving any reaction.

This stable, arranged for five cows, measured 15½ x 14½ feet, ceilings 7½ feet, two windows (one north and one south), one ventilator one foot square; average space per cow approximately 337 cubic feet.

Reviewing the history of these two cases we are confronted with a number of pregnant facts. Taking the Sloane herd these facts are:

1. That by the aid of tuberculin fourteen diseased animals were eliminated from a herd of thirty-five.

2. That eleven heifers and one young bull were all sired by a diseased bull.

3. That of these eleven heifers seven were born of diseased mothers.

4. That all of them were reared on milk, most of which was drawn from diseased cows.

5. That they never came in direct contact with the diseased animals.

6. That notwithstanding the facts that all were sired by a diseased bull, that seven of them were born of tuberculous mothers, that all of them were reared on milk from tuberculous cows, and that all of them have been kept in a stable which was previously the home of thirteen diseased cows, that notwithstanding these facts we find, at the end of four years, that at least five of these eleven animals are absolutely free from tuberculosis, and that in every human probability all of them that now remain alive are likewise free from this disease.

7. Of the cows introduced from Long Island and placed in this previously infected place some have died and been found healthy, and according to every appearance, and in accordance with every analogy, those that remain are likewise free from disease.

The lesson to be derived from the herd of Dr. Colt points exactly to the same conclusions, and yet here we must recognize a very material and important difference. In the case of Mr. Sloane, the stable was large, airy, well-lighted, ventilated, had a water-tight floor, readily lent itself to cleansing and disinfection, and was altogether, from a sanitary standpoint, well arranged.

The stable of Dr. Colt, on the other hand, was the very reverse of this. It was small, had insufficient cubic space, but little ventilation, no proper drainage, had pervious, urine-soaked floors, was finished in rough timber, and, from a sanitary point of view, was badly arranged. Taken on the whole, it is not too much to say that it was considerably worse than the average Massachusetts cow-stable. And yet, in spite of all this and the fact that it was a badly-infected stable, the evidence is that the means of disinfection were adequate and that the infection of tuberculosis was completely eradicated.

Reviewing the two cases as a whole, the immense preponderance of evidence is in favor of these conclusions:

That, by the judicious use of tuberculin, tuberculosis can be eradicated from a herd of cows.

That tuberculosis is not an hereditary disease.

That so far as calves are concerned, the danger to them from the use of milk from tuberculous cows must necessarily be very slight.

And that, given an infected building, it is possible, at comparative little cost, to make it entirely free from infection and a safe place in which to keep stock so far as the disease, tuberculosis, is concerned.

P. S.—In dealing with tuberculosis-infected buildings, with a view to rendering them aseptic and free from tubercle bacilli, there is one feature which, to my mind, has never been sufficiently appreciated by either the veterinary profession or by the laity. This feature is the necessity of dealing in the most radical and thorough manner with the floors and with the urine-soaked and feces-saturated underlying earth.

As the strength of a chain is limited to the weakness of its weakest link, so the success of prophylactic measures depends no less on their thoroughness than on their general completeness. We occasionally see water-tight floors in cow-stables, but it must be admitted that they are very exceptional. The immense majority of floors are freely pervious, and the underlying earth simply saturated with fermenting organic matters acting both as pabulum and incubator for disease-producing organisms. There are two ways by which this difficulty can be dealt with:

The starving-out process—that is, to remove all stock from the buildings, and leave them unused until the organic materials have become exhausted and the bacilli die from want of suitable food. I have known this practised with success; but it is a matter of years, a long, tedious, expensive, unscientific method.

The other and better way is to remove the floors and dig out the subjacent earth. The extent to which this has to be done depends on the nature of the soil, but it rarely happens that it is necessary to remove more than two or three feet, its place, of course, being supplied by fresh virgin earth. Nor is this and the other necessary cleaning steps an expensive process. Soap and water and manual labor are cheap, and the stables are few and far between that cannot be rendered thoroughly aseptic and wholesome for twice the cost of one good cow.

Repeated experiences of the recurrence of tuberculosis in stables where every other precaution had been rigidly enforced, but this neglected, have convinced me that, except where the floors are water-tight (as in the Sloane stable), no system of disinfection can be complete without it. And not only do I claim it is essential to completeness, but from the rapidity with which I have known clean herds to become diseased, and from the general way in which they have become diseased when no other possible means of infection could exist, I am convinced that while the necessity of dealing in a competent manner with the floors is the precaution least practised, it is, of all measures, the most essential to success.

While it is aside from the purpose I had in beginning this paper, I cannot refrain from calling attention, as in years gone by I have called attention, to the insane manner in which cows are ordinarily tied up, their heads all chained and blocked and tied together in one long trough, sneezing and coughing and hawking in each other's faces. Why not partition each cow off, if the partition be only as high as her elevated head, giving to each her own crib, where she can eat her food in peace and be to some extent protected from the infection, it may be, of her diseased neighbor?

When we remember that we are dealing with a disease which, after all, is but feebly contagious, I think that there are great possibilities for the ultimate suppression of tuberculosis along this line alone. By even mail I send you a photograph showing something of what may be done in this way. Within the past year I know of two diseased cows which had been kept for three years in such a partitioned stable along with twelve other cows without communicating the disease to them. These two cows were purchased out of a herd that was notoriously tuberculous, and were undoubtedly diseased at the time they were bought. During their three years' stay in their new quarters one cow stood at the end of the row and the other right in the middle of it.

This problem of suppressing bovine tuberculosis is engaging the attention and testing the resources of every one of the advanced civilized countries of the world. It is preëminently a problem for the veterinary profession to solve, and a task for it to work out. Along this line there are reputations to be gained and credit to be earned, and from the widespread nature of the malady there is opened up to the veterinary profession as a whole a wide field of opportunity such as seldom falls to the lot of any one profession, an opportunity to advance itself in the esteem and regard of all thinking, wide-minded people.

With tuberculin as a diagnostic agent; with powers of compulsory slaughter or isolation; and, more than all, with the enforcement of complete sanitary regulations, it seems to me a successful fight against the scourge is assured.

